# Effectiveness of a Physical Education Program on the Motor and Pre-literacy Skills of Preschoolers From the Training-To-Health Project: A Focus on Weight Status

**DOI:** 10.3389/fspor.2020.579421

**Published:** 2020-12-16

**Authors:** Giuseppe Battaglia, Valerio Giustino, Garden Tabacchi, Marianna Alesi, Claudia Galassi, Carmen Modica, Antonio Palma, Marianna Bellafiore

**Affiliations:** ^1^Department of Psychological, Educational Science and Human Movement, University of Palermo, Palermo, Italy; ^2^Sport and Exercise Science Research Unit, University of Palermo, Palermo, Italy

**Keywords:** physical education, motor skills, pre-literacy skills, preschooler, weight status

## Abstract

Many studies reported a positive relationship between motor skills, cognitive functions, and school performance in school-age children; however, little is known in preschool children. The aim of the present study was to demonstrate the effectiveness of a physical education program (PEP) on locomotor, object control skills, and pre-literacy cognitive functions in a wide population of preschoolers and verify whether weight status could influence these abilities. In the context of the Training-to-Health Project, a sample of 1,029 preschoolers was recruited in kindergartens from the urban area of Palermo (Italy). Their gross motor and pre-literacy skills were tested before (PRE) and after (POST) following 16 weeks (2 h/week) of a PEP, which included ludic-motor activities aimed at developing body awareness and fundamental motor and perceptual-sensory skills. Analyses of variance (ANOVA) were performed to assess the skills before and after the intervention and to evaluate the effect of different categories of weight status on the examined variables. Regression analyses were conducted to confirm the hypothesized interrelationship between motor and pre-literacy skills in the considered sample. Both locomotor/object control and pre-literacy skills were significantly higher in children after the PEP (*p* < 0.05). We found 23% of overweight children and no significant difference between weight status classes in both PRE and POST PEP groups. In the POST group, higher locomotor and object control skills were mostly associated with better pre-literacy skills. This study shows that PEP was effective in improving both motor and pre-literacy skills in preschoolers independently from age and gender, while weight status did not affect these skills suggesting that this program can be administrated indifferently in children with different categories of weight status. Therefore, PEP could be a decisive education strategy to enhance motor and cognitive learning in preschool children and to achieve successful academic outcomes.

## Introduction

### Motor Skills

Fundamental movement skills (FMS), or simply motor skills, are defined as the basic level of movement and include locomotor skills (running, jumping, galloping, hopping, crawling) and object control (bouncing, catching, throwing, kicking) (Bellows et al., 2017; Han et al., 2018). The concept of FMS is strictly related to the quality of motor skill competence (MC), which is the level ability of motor coordination, and it is measured through several test batteries (D'Hondt et al., 2009; Lopes et al., 2012; Figueroa and An, 2017; Maeng et al., 2017; Loras, 2020; Tsuda et al., 2020).

The appropriate development of FMS, which occurs during the preschool period (children aged 3–5 years), allows to build the basis for performing complex movements and task-specific abilities (Figueroa and An, 2017; Aivazidis et al., 2019; Alves and Alves, 2019). Moreover, several studies reported that the practice of physical activity (PA) improves FMS level by inducing a higher MC in childhood (Fisher et al., 2005; Hardy et al., 2010; Battaglia et al., 2018; Aivazidis et al., 2019; Nilsen et al., 2020a). For these reasons, the practice of structured PA in kindergarten during this period results to be crucial (Deli et al., 2006; Barbosa et al., 2016; Figueroa and An, 2017; Kippe and Lagestad, 2018; Alves and Alves, 2019; Jarraya et al., 2019). However, PA in preschool age is limited and, furthermore, the level of PA among children living in Europe varied considerably among countries (Konstabel et al., 2014). The latter topic is also confirmed by the fact that over the years several research groups have focused on the importance of PA in children and adolescents, and the few articles present in the literature about this issue in preschooler demonstrate the recent interest among scientists (Van Capelle et al., 2017; Engel et al., 2018).

It is well-known that PA plays a key role in promoting health and preventing diseases at every stage of life, including children and adolescents (Nielsen et al., 2016; Voss and Harris, 2017; Czenczek-Lewandowska et al., 2018). The lack/low level of PA practice and sedentary behaviors negatively affect body weight in preschooler children (Han et al., 2010; Osinski and Kantanista, 2017; Rodriguez-Ayllon et al., 2019). Furthermore, the assumption of harmful behaviors (e.g., eating unhealthy foods) during the time spent in a sedentary lifestyle has been shown to exacerbate the capacity to maintain an adequate body weight in children by creating a vicious cycle (Pagani et al., 2010; Eichinger et al., 2017). As reported by several researches, both physical inactivity and sedentary/unhealthy behaviors increase the risk of being overweight and obesity in childhood (Tucker, 2008; Han et al., 2010; Hills et al., 2011; Reilly et al., 2019). For this reason, the role of diet and physical activity in children has been investigated in previous studies (Obradovic Salcin et al., 2019; Tabacchi et al., 2020a). In support of the above, Tabacchi et al. (2020a) suggest that raising children in an environment where both motor and cognitive skills are encouraged can improve food literacy and, furthermore, enhance school achievement.

The prevalence rate of overweight and obesity among European children, with different rates between countries, is about 20% and represents a public health problem due to the related risk factors (Ahrens et al., 2014; Eichinger et al., 2017). In order to contrast this phenomenon, a series of political and social initiatives and experimental approaches have been promoted by encouraging the practice of PA among preschoolers (Palmer et al., 2019; Hoza et al., 2020; Popovic et al., 2020).

The beneficial effects of PA for preventing overweight and obesity are well-documented. Furthermore, as reported in the review by Bingham et al. (2016), the interrelationship between PA and several socio-ecological domains, such as anthropometric/demographic features (e.g., age, gender, weight status), and family community level/parental behaviors (e.g., family socioeconomic status/parents' PA level) is widely recognized in early children (Kimbro et al., 2011; Eichinger et al., 2017). In a similar way, FMS have reported to positively affect psychosocial aspects and cognitive functions in preschool-aged children (Rhemtulla and Tucker-Drob, 2011; Oberer et al., 2017). However, only a few studies have investigated the influence of motor skills on PA levels and preschoolers' body weight status, reporting conflicting outcomes (D'Hondt et al., 2009; Logan et al., 2011; Morano et al., 2011; Lopes et al., 2012; Roscoe et al., 2019).

### Pre-literacy Skills

Positive influence of PA programs on pre-literacy skills has been found in the kindergarten age (Barnett et al., 2008; Callcott et al., 2015; Mavilidi et al., 2015, 2017).

Pre-literacy refers to kindergarten skills that are predictors of later literacy achievement. These skills include a set of competences, such as (1) knowledge and understanding about printed materials; (2) oral language abilities, e.g., vocabulary, comprehension, and listening; and (3) alphabetic code awareness, e.g., phonological/phonemic abilities to detect and manipulate syllables, phonemes, and word parts (Lonigan and Shanahan, 2010; Puranik and Lonigan, 2011; Pinto et al., 2016).

Given these positive influences of PA on pre-reading/writing and pre-math, researchers and practitioners have developed a variety of preschool-based programs suitable to enhance pre-literacy skills through PA activities. The underlying conceptualization is that active play characterizing preschool-age PA would be a natural and enjoyable context to encourage linguistic development by creating more frequent linguistic and social opportunities, to understand and explain instructions or play rules, to elaborate stimuli from many sources, to experiment direct or indirect performances, and to try new action-based representations of tasks (Boncoddo et al., 2010; Carson et al., 2015).

For example, the Movement with Literacy (MowL) was found to improve phonological awareness, spelling, and motor coordination. It was a program based on a 15-min daily preschool curriculum composed of literacy (Let's Decode) and movement (Moving on with Literacy) curricula (Callcott et al., 2015) and counted in 30 action songs with motor tasks to train fine and gross motor, eye-tracking, balance, rhythm, core strength, and aerobic skills. Another program delivered by classroom teachers over 8 months was made up of 60-min moderate PA units (2 times per day) associating motor (jumping, running, moving on lines, marching) and early literacy tasks to train oral language, vocabulary, and phonological awareness (Kirk et al., 2014; Kirk and Kirk, 2016). Programs implemented by Mavilidi et al. (2015, 2017) improved phonological and science competences in preschool age. Rhyming, alliteration, and picture naming skills were enhanced through PA activities that associated full-body movements, e.g., physical exercises, or part-body movements, e.g., gestures, and correspondent foreign words. Science competences were improved by an integrated method that trained children to learn planets' names and their correct position from the sun by performing movements from the sun to the closest planet and so on.

Moreover, a Parent-oriented Movement and Pre-literacy Program required 60 min a week over 10 weeks of activities and involved both preschool age children and their parents. It consisted of Fundamental Movement tasks; free-play activities with balls, steps, bricks, or puzzles; and a storybook reading activity shared among children and their parents to enhance motor and literacy skills as print-concept and alphabet knowledge (Bedard et al., 2017).

### Purpose

In our previous work we investigated the effect of a 16-weeks physical education program (PEP) in motor and cognitive preschool children's status, finding an improvement on both domains (i.e., motor skills and cognitive functions) (Battaglia et al., 2018). However, as stated in that paper, in the present study we applied the further step for the validation of the study, i.e., to extend the investigation to a larger sample size in order to illustrate the effects of the developed PEP on locomotor, object control skills, and pre-literacy cognitive functions in a larger scale (Battaglia et al., 2018). Moreover, based on this large preschooler sample, the further purpose of the study was to investigate whether weight status could influence these skills.

## Materials and Methods

### Study Design and Participants

The present study is a non-randomized trial carried out within the Training-to-Health Project, financed by the Municipality of Palermo in 2016 with the general aim of enhancing motor and cognitive skills in preschoolers, beyond other scopes, such as monitoring aspects related to these abilities (Tabacchi et al., 2020b). A team of experts from the University conducted the study and recruited physical education specialists (PESs) with previous experience in the field of motor and cognitive science in children, in order to carry out activities within the selected kindergartens. Teachers from the classes were directly involved as support in the program activities. All the personnel taking part in the program was properly trained, and methodologies were standardized to allow the collection of accurate and reliable data.

The study was approved by the Ethical Board of the University of Palermo (N. 2/2018) and followed the criteria for the use of persons in research as defined in the Declaration of Helsinki.

The preschoolers' sampling envisaged a mixed multistage procedure. In the first stage, all the kindergartens of Palermo city boundaries (Municipality of Palermo) (*n* = 389) were identified; in the second stage, the sample was stratified into public (*n* = 193) and private kindergartens (*n* = 196) and the cluster of public kindergartens was selected; in the third stage, *n* = 21 (11%) public kindergartens were randomly chosen in geographical areas stratified by different socioeconomic environments (SEE), every class within the chosen cluster was chosen, and every student within the class was sampled (*n* = 1,054). The SEE was denoted according to the “index of socio-economic disadvantage,” measured on the basis of four indicators of deprivation in the 55 different city districts (http://cqd.comune.palermo.it/CQDSupera/docs/CQD4982_1221223025117.pdf).

Parents were asked to sign an informed consent to let their children participate the study. Among them, a total of 25 refused to sign (2.4%), and a final sample of 1,029 children was obtained.

Since collecting measures for all the individuals was not possible, as some of them were not present the day of the assessment of a particular item, some results are referred to a slightly lower sample, as indicated in the result tables.

### Study Procedure

Participants' weight and height were initially measured, and they were assessed for basic motor skills and pre-literacy abilities by the PESs (we will further indicate this group as the “PRE group”).

The motor skill items were measured by the Italian version of the gross motor development test (Ulrich, 2003), which consists in two different aspects of gross motor development, i.e., locomotion (requiring subjects to run as fast as possible for 15 m, jump forward, gallop for 10 m, hop on one leg for 5 m, do a long jump, and take little jumps forward and laterally), and object control (bounce the ball, catch the ball, catch a ball with a tennis racket, and running while kicking a ball and throwing a ball). The combination of these two subtests provides the quotient of gross motor development (QGMD), useful for assessing the overall gross motor skills of children.

Pre-literacy abilities were assessed by choosing four tasks related to visual analysis and spatial orientation abilities, derived from the Italian battery PRCR-2/2009 (Cornoldi et al., 2009), measuring general and specific prerequisites to reading and writing abilities in preschoolers.

Details of these assessment tools are described in our previous paper (Battaglia et al., 2018).

After the initial assessment, preschoolers were subjected to a Physical Education Program (PEP) for 16 weeks, applied with a frequency of twice a week, by the PESs. In agreement with Battaglia's PEP (Battaglia et al., 2018), each lesson (~60 min) included a warm-up and social interaction phase (~5 min) which aimed to enhance the fitness level of children and their motivation to participate in activities (e.g., circle time in which children, sitting on the floor, greeted everyone and took turns performing a movement requested by the physical education specialist; activities of running, jumping, catching/throwing/kicking a ball, etc.); a central phase (~50 min) including specific activities in order to develop perceptual-sensory and fundamental motor and skills in preschool children; and a cool-down phase (~5 min) in order to relax children and explore their level of satisfaction (e.g., circle time in which children, lying on the ground, performed calming breathing activities). At the end of the PEP, preschoolers were assessed again for the gross motor and pre-literacy skills, and also anthropometric measures were collected again. This group following the PEP represents the “POST group.” Children of the PRE group were included in classroom activities for an equal time as the POST group with teachers. Both groups completed the activities during the school period in a multi-activity area.

### BMI and Motor Skill Measures

Age of the preschoolers was retrieved from the birth dates reported in the kindergartens' lists.

Weight and height were measured through Seca electronic scales (maximum weight recordable, 300 kg; resolution, 100 g; Seca Deutschland, Hamburg, Germany) and stadiometers (maximum height recordable, 220 cm; resolution, 1 mm) which were used according to standard procedures (Lohman et al., 1988). Body mass index (BMI) was calculated, and four classes of weight status were obtained according to the BMI percentiles for males and females aged 0–18 years from Cole et al. (2000): underweight below the 5th percentile, normal weight between the 5th and 85th percentiles, overweight between the 85th and 95th percentiles, and obese over the 95th percentile.

The gross motor skills were measured as scores derived from the marks attributed to the single performance. All participants completed three trials of each gross-motor skill and acquired a “1” mark, when a criterion was used incorrectly two out of three times or was not observed, or a “0” grade, when a criterion performance was executed two out of three times. According to the age level of the participant, the sum of the scores detected for each item (maximum total score 48) was converted into standard scores. The QMGD was obtained by summing up the scores of the two subtests for assessing locomotor and object control skills, and it could score from 46 to 154.

The categorization of QGMD in classes of motor abilities was performed according to the manual (Ulrich, 2003): 35–69 (very low motor ability, VL-MA), 70–79 (low motor ability, L-MA), 80–89 (below average motor ability, UA-MA), 90–110 (average motor ability, A-MA), 111–120 (over average motor ability, OA-MA), 121–130 (high motor ability, H-MA), and 131–165 (very high motor ability, VH-MA).

### Pre-literacy Skill Measures

Pre-literacy skills were measured through four tasks derived by PRCR-2/2009 (Cornoldi et al., 2009) which is an Italian battery of standardized tasks to test kindergarten children's prerequisites to later reading and writing abilities. The tasks were (1) printed-letter identification aimed at measuring visual analysis and spatial orientation abilities; (2) object naming aimed at measuring linguistic proficiency, the visual attention, and the sequentiality of eye movements; (3) partially hidden-object naming aimed at measuring linguistic proficiency, the visual attention and discrimination, and the sequentiality of eye movements; and (4) pointed-object naming aimed at measuring the visuo-perceptual ability to identify a figure from the background, the linguistic proficiency, the visual attention and discrimination, and the sequentiality of eye movements. More in detail, the printed-letter identification task consisted of a white sheet with 12 target letters printed on the left and four letters for each target (the target and three distracting letters) printed on the right. Each child was asked to identify and cross the letter corresponding to the right target. The object-naming task was composed of 30 objects in five sequences of six objects for each (e.g., animals, flowers, ice cream, sun, star), and each child was asked to name each object. The partially-hidden-object naming task was composed of three sequences of objects, already shown in the object-naming task, but the objects were overlapping and smaller. Each child was asked to recognize and name each object. The pointed-object-naming task was composed of two sequences of overlapping objects that appeared in the partially hidden-object-naming task with four objects for each sequence marked by a dot at 15 mm. Each child was asked to rapidly name the marked objects from left to right and from top to bottom.

The criteria of evaluation were the sum of errors and the time of performance.

### Statistical Analysis

Sample descriptive data are shown in number and percentages for categorical outcomes and mean and Standard Deviation (SD) for normally distributed continuous data. Normality of data was evaluated through the skewness/kurtosis test for normality.

The difference between PRE and POST groups skills was calculated through analysis of variance (ANOVA) adjusted for age and gender. This analysis was also used to assess differences in gross motor skills and pre-literacy abilities by classes of weight status.

Linear regression analyses were conducted to assess the degree of correlation between gross motor and pre-literacy abilities. Significance was set at *p* < 0.05. The software STATA.12 was used to perform the statistical analyses.

## Results

### Sample Characteristics

The characteristics of the sample are shown in Table 1.

**Table 1:** Characteristics of the sample before (PRE) and after (POST) the physical education program.

	**No**.	**%**		
**Gender**				
Males	557	54.1		
Females	472	45.9		
Tot	1,029	100		
	**Pre**		**Post**	
**Age**	**No**.	**%**	**No**.	**%**
3 years (≤47 months)	200	19.4	142	13.8
4 years (48–59 months)	393	38.2	380	36.9
5 years (≥60 months)	436	42.4	507	49.3
Tot	1,029	100	1,029	100
**Weight status**				
Normal	587	69.5	555	66.5
Under	90	10.7	87	10.4
Over	127	15.0	130	15.6
Obese	41	4.9	62	7.4
Tot	845	100	834	100
	**Pre**		**Post**	
	**Mean**	**SD**	**Mean**	**SD**
Age (months)	56.11	9.85	58.98	10.03
Height (m)	1.08	0.07	1.09	0.08
Weight (kg)	19.05	3.93	19.36	4.13
BMI (kg/m^2^)	15.94	2.02	16.07	2.31

### Differences Between PRE and POST PEP

The analysis of variance showed that the QGMD was significantly higher in the POST group compared to the PRE group (mean 108.8 vs. 123.9, *p* < 0.001); according to the QGMD classes, it passes from “average motor ability” in the PRE group to “high motor ability” in the POST group. This high difference was present also for the components of the QGMD, i.e., locomotor (mean 10.7 vs. 14.0) and object control skills (mean 12.3 vs. 14.1) (Table 2). Moreover, all the evaluated locomotor and object control subitems were significantly higher in the test group (Table 2), with an average score increase of 0.63 units (range 0.5–0.8) and 0.55 units (range 0.47–0.7), respectively.

**Table 2:** Evaluation of the locomotor and object control skills before and after the physical education program.

	**PRE**		**POST**		**N**	*p*-Value^*^
	**Mean**	**SE**	**Mean**	**SE**		
**QGMD**	108.8	0.78	123.9	0.62	805	0.0000
**Locomotor skills**	10.7	0.17	14.0	0.15	807	0.0000
***Locomotor subitems***						0.0000
Running	2.6	0.04	3.2	0.04	966	0.0000
Galloping	2.0	0.05	2.7	0.05	967	0.0000
Hopping	1.7	0.05	2.5	0.05	967	0.0000
Leaping	1.2	0.04	1.7	0.04	967	0.0000
Horizontal jumping	2.2	0.04	2.9	0.05	967	0.0000
Skipping	1.3	0.04	1.8	0.04	967	0.0000
Sliding	2.3	0.05	2.9	0.05	967	0.0000
**Object control skills**	12.3	0.14	14.1	0.15	966	0.0000
***Object control subitems***						
Two-hand striking	1.4	0.04	2.1	0.05	967	0.0000
Stationary bouncing	1.03	0.03	1.5	0.04	967	0.0000
Catching	2.2	0.04	2.8	0.05	967	0.0000
Kicking	2.0	0.04	2.5	0.05	967	0.0000
Overhand throwing	1.9	0.05	2.4	0.05	966	0.0000

*QGMD, Quotient of Gross Motor Development*.

* *The difference between pre and post was calculated through ANOVA adjusted for age and gender. Significance set at p < 0.05*.

With regard to the pre-literacy skills, all of them significantly increased in the POST PEP group, meaning that both errors and time needed to perform the test were significantly lower in this group compared to the PRE group (Table 3). For example, the errors in the identification of printed letters were on average 3.4 in the PRE group and they decreased to 2.7 in the POST group; the time needed to naming objects decreased from 72.8 to 62.2 s. On average, the number of errors decreased by 1.05 units (range from 0.7 to 2 units), while the time needed in naming objects decreased of 14.5 s (10.6 and 18.4 s in naming objects and naming partially hidden objects, respectively).

**Table 3:** Evaluation of the pre-literacy skills before and after the physical education program.

	**PRE**		**POST**		***N***	*p*-Value^*^
	**Mean**	**SE**	**Mean**	**SE**		
Printed-letter identification (error n.)	3.4	0.10	2.7	0.10	751	0.0000
Object naming (s)	72.8	1.09	62.2	0.89	713	0.0000
Object naming (error n.)	2.0	0.09	1.2	0.07	709	0.0000
Partially-hidden-object naming (s)	116.6	1.85	98.2	1.37	688	0.0000
Partially-hidden-object naming (error n.)	5.6	0.20	3.6	0.16	730	0.0000
Pointed-object naming (error n.)	2.3	0.06	1.6	0.06	698	0.0000

* *The difference between pre and post was calculated through ANOVA adjusted for age, gender. Significance set at p < 0.05*.

### Differences by Weight Classes

When analysis of variance was conducted by categories of weight status, the QMGD, locomotor, and object control skills were not significantly different throughout the classes of underweight, normal overweight, and obesity (Table 4). Actually, observing all the skill values in obese preschoolers, reduced performances can be evidenced, even though they were not statistically significant. The same observation can be done for the pre-literacy skills, with a trend of decreasing performances from normal weight to obese individuals (Table 5) but no statistical significance in those differences. Similar results were found out also for all the locomotor subitems, with the exception of leaping (*p* < 0.05), and for all the object control subitems (Supplementary Material 1).

**Table 4:** Differences in QMGD, locomotor, and object control skills by categories of weight status.

	**QMGD**			**Locomotor skills**			**Object control skills**		
	**Mean**	**SD**	***p*-Value**	**Mean**	**SD**	***p*-Value**	**Mean**	**SD**	***p*-Value**
Weight status			0.0843			0.3247			0.0578
Normal	123.0	17.22		13.5	3.73		14.3	2.86	
Under	125.6	16.92		13.8	3.43		14.7	2.71	
Over	124.3	18.46		13.8	3.79		14.4	3.20	
Obese	117.2	19.93		12.6	3.91		13.2	2.41	

**Table 5:** Differences in pre-literacy skills by categories of weight status.

	**Printed-letter identification (error n.)**			**Object naming (s)**			**Object naming (error n.)**		
	**Mean**	**SD**	***p*-Value**	**Mean**	**SD**	***p*-Value**	**Mean**	**SD**	***p*-Value**
**(A) Printed letters identification and objects naming**									
Weight status			0.1593			0.1078			0.6410
Normal	3.0	2.98		63.0	23.89		1.3	1.06	
Under	2.36	2.32		68.4	28.23		1.1	0.63	
Over	2.55	1.68		62.9	27.73		1.1	0.63	
Obese	1.75	1.58		55.0	25.35		0.9	0.68	
	**Partially-hidden-object naming (s)**			**Partially-hidden-object naming (error n.)**			**Pointed-object naming (error n.)**		
	**Mean**	**SD**	***p*****-Value**	**Mean**	**SD**	***p*****-Value**	**Mean**	**SD**	***p*****-Value**
**(B) Partially-hidden-object naming and pointed-object naming**									
Weight status			0.3365			0.7230			0.9670
Normal	101.4	36.53		3.8	4.67		1.61	1.45	
Under	107.6	45.72		3.4	3.28		1.6	1.47	
Over	100.6	32.93		3.5	4.85		1.63	1.56	
Obese	90.2	39.79		2.9	3.78		1.56	1.55	

These results were found out both in POST and PRE PEP groups (PRE group results are not shown in the present paper).

### Regressions of Motor and Pre-literacy Skills (POST)—Adjusted for Age and Gender

The regression analysis showed that children with high QGMD make less errors in naming objects and partially hidden objects (coefficients −1.22 and −0.49) and take less time in naming pointed objects (coefficients −0.55) (Table 6). The component of object control skills is significantly correlated with all the pre-literacy skills, with higher object control ability associated with better pre-literacy ability (coefficients ranging from −0.04 to −1.13); for the locomotor skills, the time needed in performances was not correlated, but all the other items (errors) were with coefficients ranging from −0.16 to −0.86 (Table 6).

**Table 6:** Results of the regression analysis between motor and pre-literacy skills.

	**QGMD**				**Locomotor skills**				**Object-control skills**			
	**Coef**	**SE**	***p***	***N***	**Coef**	**SE**	***p***	***N***	**Coef**	**SE**	***p***	***N***
Printed letters	−0.25	0.248	0.324	707	−0.28	0.087	*0.001*	743	−0.18	0.064	*0.005*	744
Object naming time (s)	−0.03	0.031	0.355	673	−0.04	0.011	*0.000*	708	−0.004	0.008	0.576	709
Object-naming errors (n.)	−1.22	0.377	*0.001*	666	−0.59	0.129	*0.000*	702	−0.32	0.093	*0.001*	702
Partially-hidden-object-naming time (s)	−0.03	0.019	0.136	666	−0.02	0.007	*0.008*	701	−0.001	0.005	0.818	701
Partially-hidden-object-naming errors (n.)	−0.49	0.138	*0.000*	698	−0.25	0.048	*0.000*	734	−0.16	0.035	*0.000*	734
Pointed-object naming	−0.55	0.453	*0.000*	679	−1.13	0.157	*0.000*	715	−0.86	0.114	*0.000*	715

*The italic was used to underline the significant values (p < 0.05)*.

These results are confirmed when analyzing the locomotor and object control subitems (Supplementary Material 2).

## Discussion

The aim of the present study was to investigate the effect of a physical education program (PEP) on motor and cognitive skills in a large preschooler sample focusing on the influence of children's weight status.

As we hypothesized, our findings showed an improvement of the QGMD level and in both of its components, i.e., locomotor and object control skills, after the PEP. These results are in agreement with the outcomes we found in our previous pilot trial in which we examined the effectiveness of this PEP on the aforementioned skills in a sample of 119 preschool children (Battaglia et al., 2018). Since, as we reported in that work, the main issue was to extend the PEP on a larger scale in order to validate its effectiveness, the present study allowed us to confirm previous results.

Our findings are consistent with scientific evidence from the literature demonstrating the positive effect of PA on improving motor and pre-literacy skills in preschool age (Orton et al., 2009; Logan et al., 2012; Zeng et al., 2017; Aivazidis et al., 2019; Popovic et al., 2020). Indeed, although the literature regarding the practice of PA in kindergarten is limited, recent researches have investigated the effects of different PA interventions on several skills and health aspects in preschoolers (Popovic et al., 2020; Toussaint et al., 2020). In 2008, Stodden et al. developed a conceptual model hypothesizing a primary connection between the level of PA and the quality of MC (Stodden et al., 2008). The findings of this seminal work have been confirmed by several subsequent research groups (Lai et al., 2014; Engel et al., 2018; Nilsen et al., 2020b; Xin et al., 2020). Goodway et al. (2003) showed the positive effects of a 9-weeks integrative PA program (18 lessons, 35/min each) on the Test of Gross Motor Development (TGMD) scores in preschoolers compared to children who only carried out the regular kindergarten PA. Based on these results, the authors supported the idea of implementing PA in the kindergarten education program (Goodway et al., 2003). Likewise, DuBose et al. (2018) found a positive relationship between the level of PA in children, measured though an accelerometer, and motor skills. In the same way, pre-literacy skills in preschoolers are positively related to PA programs (Carson et al., 2016; Donnelly et al., 2016; Zeng et al., 2017).

As abovementioned, the results we found regarding the effect of PA on motor and pre-literacy skills are in line with several authors (Callcott et al., 2015; Mavilidi et al., 2015, 2017; Kirk and Kirk, 2016; Bedard et al., 2017). Children with high QGMD performed better on PRCR-2 tasks. These results revealed in these children a higher level on the abilities of visual analysis, visual attention, visual discrimination, spatial orientation, and linguistic proficiency. This could be due to PEP that should enable kindergarten children to train visuospatial abilities, which are necessary to master print directionality, to align or put in a column the numbers with consequent accuracy on computation and numerical representations. Visual-motor integration abilities are essential to organizing and spacing letters, words, and numbers on a page. Moreover, motor tasks as jumping, running, moving on lines, and marching revealed to be suitable to train the ability to recognize and produce alliteration and rhyming (Mavilidi et al., 2015, 2017; Kirk and Kirk, 2016). In the context of interaction with peers and movement exercises based on rhythm, fine and gross motor practice, eye-tracking, and balance, children have more opportunities to develop their linguistic and metalinguistic skills, such as alphabet knowledge, letter–sound knowledge, detection and manipulation of phonemes or syllables, and the knowledge to encode and decode words.

While when the effects of PA were analyzed by weight status categories, no significant differences were found on the QMGD and locomotor and object control skills between underweight, normal overweight, and obesity, in both PRE and POST PEP groups. Although few studies have investigated the relationship between motor skills and weight status/BMI domain in preschool age, the findings are conflicting (Lopes et al., 2012; Kim and Lee, 2016; Augustijn et al., 2018; Roscoe et al., 2019). For instance, D'Hondt et al. (2009) showed significantly higher levels of balance, object control, and manual dexterity in children with normal weight and overweight than obese peers. Similarly, Logan et al. (2011) reported significant differences on MC among children ages 4–6 years with a different BMI status indicating lower scores of motor skill proficiency in overweight and obese children compared to normal-weight peers. In contrast, the results reported by a large body of increasing research showed no correlation between FMS and weight status/BMI levels in preschoolers, as we found (Saraiva et al., 2013; Kim and Lee, 2016; Mülazimoglu Balli, 2016).

The key explanation could lie in the fact that cortical areas reach different levels of maturity of each other during the development process, and, moreover, the relationship between executive functioning and weight status in children has only been discovered in some cerebral areas (Liang et al., 2014). Among the latter, the literature suggests that at an early age the prefrontal cortex results are less developed and this physiological condition does not allow to identify any differences in motor and pre-literacy skills among preschoolers belonging to distinct weight classes (Liang et al., 2014).

Although we found no difference between weight status groups, our results showed lower scores in leaping in children classified as overweight/obese. We suppose that weight status adversely affects this skill performance. Our hypothesis is sustained by several researches in which it has been demonstrated that tasks requiring propulsion are strictly dependent on body mass, reporting an inverse relationship between the amount of body mass and the lower-limb strength scores, which is an association between motor skills and overweight/obese status only for some locomotor skills (Siahkouhian et al., 2011; Castetbon and Andreyeva, 2012).

Therefore, our outcomes seem to suggest that this PEP can be administered in preschoolers with different weight status categories likewise, in order to improve motor and cognitive skills and to provide favorable bases for enabling positive academic achievement.

In the conceptual model of Stodden et al. (2008), an association between higher level of PA and greater scores in both locomotor and object control skills has been suggested. Moreover, the authors support the notion that preschool children with poor level of FMS could be more physically inactive in the future, leading, among other things, to an increased risk of being overweight/obese (Stodden et al., 2008).

As preschoolers currently perform lower than recommended PA levels by increasing the associated health risk and compromising skill development and later school achievement, we suggest that, based on our results, children would spend more time in structured PA in kindergartens (Gu, 2016; Kippe and Lagestad, 2018; Kobel et al., 2019). Therefore, we suggest that this PEP could be adopted as an educational strategy to improve motor and pre-literacy skills in preschool children, known to be a peculiar growth period for the development of these characteristics. Furthermore, we suggest that the activities of this PEP can also be proposed by teachers online, a modality that could be useful in critical and peculiar periods in which the level of physical activity could decrease as during the current COVID-19 pandemic (An, 2020; Giustino et al., 2020). To sum up, the implementation of enjoyable methods of teaching to enhance children's school readiness through motor activities is an important matter to be investigated by educational scientists.

## Data Availability Statement

The raw data supporting the conclusions of this article will be made available by the authors, without undue reservation.

## Ethics Statement

The studies involving human participants were reviewed and approved by Ethical Board of the Azienda Ospedaliera Universitaria Policlinico Paolo Giaccone Palermo (Palermo 1, N. 02/2018). Written informed consent to participate in this study was provided by the participants' legal guardian/next of kin.

## Author Contributions

MB conceptualized the paper. CG and CM collected the data. GT carried out the formal curation and analysis of data and drafted the results. GB, VG, and GT drafted original draft. MB, MA, and GB reviewed and edited the manuscript. AP supervised the final manuscript. All authors contributed to the article and approved the submitted version.

## Conflict of Interest

The authors declare that the research was conducted in the absence of any commercial or financial relationships that could be construed as a potential conflict of interest.
