# Supplementary material for: Effectiveness of a Physical Education Program on the Motor and Pre-literacy Skills of Preschoolers From the Training-To-Health Project: A Focus on Weight Status
Source: Front Sports Act Living. 2020 Dec 16;2:579421. doi: 10.3389/fspor.2020.579421 (PMC7750875; doi:10.3389/fspor.2020.579421)
Supplement: Supplementary file 1 [file Data_Sheet_1.docx]

**Supplementary Material 1.**

**Table S1.** Differences in locomotor sub-items skills by categories of weight status.

|  | **Running** | | | **Galopping** | | | **Hopping** | | | **Leaping** | | | **Horizontal jumping** | | | **Skipping** | | | **Sliding** | | |
| --- | --- | --- | --- | --- | --- | --- | --- | --- | --- | --- | --- | --- | --- | --- | --- | --- | --- | --- | --- | --- | --- |
|  | mean | SD | p-value | mean | SD | p-value | mean | SD | p-value | mean | SD | p-value | mean | SD | p-value | mean | SD | p-value | mean | SD | p-value |
| ***Weight status*** |  |  | 0.5087 |  |  | 0.0815 |  |  | 0.6790 |  |  | *0.0412* |  |  | 0.0613 |  |  | 0.1263 |  |  | 0.3437 |
| **normal** | 3.3 | 1.18 |  | 2.8 | 1.31 |  | 2.6 | 1.45 |  | 1.8 | 1.15 |  | 3.0 | 1.27 |  | 1.9 | 1.13 |  | 3.0 | 1.30 |  |
| **under** | 3.2 | 1.34 |  | 2.6 | 1.44 |  | 2.5 | 1.45 |  | 1.9 | 1.02 |  | 2.9 | 1.28 |  | 2.0 | 1.12 |  | 2.8 | 1.42 |  |
| **over** | 3.2 | 1.22 |  | 3.0 | 1.26 |  | 2.6 | 1.38 |  | 2.0 | 1.07 |  | 3.0 | 1.24 |  | 2.0 | 1.15 |  | 2.9 | 1.34 |  |
| **obese** | 3.1 | 1.25 |  | 2.8 | 1.31 |  | 2.7 | 1.33 |  | 1.6 | 1.10 |  | 2.6 | 1.31 |  | 1.7 | 1.15 |  | 3.0 | 1.29 |  |

**Table S2.** Differences in object control sub-items skills by categories of weight status.

|  | **Two-hand striking** | | | **Stationary bouncing** | | | **Catching** | | | **Kicking** | | | **Overhand throwing** | | |
| --- | --- | --- | --- | --- | --- | --- | --- | --- | --- | --- | --- | --- | --- | --- | --- |
|  | mean | SD | p-value | mean | SD | p-value | mean | SD | p-value | mean | SD | p-value | mean | SD | p-value |
| ***Weight status*** |  |  | 0.1776 |  |  | 0.1764 |  |  | 0.1019 |  |  | 0.3196 |  |  | 0.7801 |
| **normal** | 2.2 | 1.35 |  | 1.6 | 1.16 |  | 3.0 | 1.26 |  | 2.7 | 1.35 |  | 2.5 | 1.40 |  |
| **under** | 2.2 | 1.38 |  | 1.8 | 1.13 |  | 2.9 | 1.43 |  | 2.6 | 1.33 |  | 2.4 | 1.43 |  |
| **over** | 2.5 | 1.28 |  | 1.8 | 1.14 |  | 3.0 | 1.31 |  | 2.7 | 1.31 |  | 2.6 | 1.41 |  |
| **obese** | 2.2 | 1.30 |  | 1.6 | 1.14 |  | 2.7 | 1.29 |  | 2.4 | 1.43 |  | 2.5 | 1.47 |  |
