# Supplementary material for: Effectiveness of a Physical Education Program on the Motor and Pre-literacy Skills of Preschoolers From the Training-To-Health Project: A Focus on Weight Status
Source: Front Sports Act Living. 2020 Dec 16;2:579421. doi: 10.3389/fspor.2020.579421 (PMC7750875; doi:10.3389/fspor.2020.579421)
Supplement: Supplementary file 2 [file Data_Sheet_2.docx]

**Supplementary Material 2**

**Table S3.** Results of the regression analysis between locomotor skills sub-items and pre-literacy skills

|  | **Running** | |  |  | **Galopping** | |  |  | **Hopping** | |  |  |  |  |  |  |
| --- | --- | --- | --- | --- | --- | --- | --- | --- | --- | --- | --- | --- | --- | --- | --- | --- |
|  | coef | SE | p | N | coef | SE | p | N | coef | SE | p | N |  |  |  |  |
| **PRINTED LETTERS** | -0.05 | 0.015 | *0.001* | 772 | -0.04 | 0.017 | *0.010* | 772 | -0.07 | 0.018 | *0.000* | 772 |  |  |  |  |
| **OBJECT NAMING TIME (sec)** | -0.007 | 0.002 | *0.000* | 733 | -0.01 | 0.002 | *0.006* | 733 | -0.01 | 0.002 | *0.000* | 733 |  |  |  |  |
| **OBJECT NAMING ERRORS (n.)** | -0.08 | 0.023 | *0.001* | 730 | -0.06 | 0.025 | *0.018* | 730 | -0.08 | 0.027 | *0.003* | 730 |  |  |  |  |
| **PARTIALLY HIDDEN OBJECT NAMING TIME (sec)** | -0.003 | 0.001 | *0.004* | 725 | 0.001 | 0.001 | 0.476 | 725 | -0.05 | 0.001 | *0.001* | 725 |  |  |  |  |
| **PARTIALLY HIDDEN OBJECT NAMING ERRORS (n.)** | -0.02 | 0.009 | *0.015* | 762 | -0.02 | 0.009 | *0.023* | 762 | -0.06 | 0.010 | *0.000* | 762 |  |  |  |  |
| **POINTED OBJECTS NAMING** | -0.16 | 0.028 | *0.000* | 743 | -0.14 | 0.03 | *0.000* | 743 | -0.21 | 0.033 | *0.000* | 743 |  |  |  |  |
|  |  |  |  |  |  |  |  |  |  |  |  |  |  |  |  |  |
|  | **Leaping** | |  |  | **Horizontal jumping** | | |  | **Skipping** | |  |  | **Sliding** |  |  |  |
|  | coef | SE | p | N | coef | SE | p | N | coef | SE | p | N | coef | SE | p | N |
| **PRINTED LETTERS** | -0.07 | 0.014 | *0.000* | 772 | -0.05 | 0.016 | *0.002* | 772 | -0.06 | 0.015 | *0.000* | 772 | -0.06 | 0.016 | *0.000* | 772 |
| **OBJECT NAMING TIME (sec)** | -0.05 | 0.002 | *0.008* | 733 | -0.01 | 0.002 | *0.000* | 733 | -0.04 | 0.002 | *0.027* | 733 | -0.05 | 0.002 | *0.004* | 733 |
| **OBJECT NAMING ERRORS (n.)** | -0.09 | 0.021 | *0.000* | 730 | -0.09 | 0.024 | *0.000* | 730 | -0.07 | 0.022 | *0.001* | 730 | -0.08 | 0.025 | *0.001* | 730 |
| **PARTIALLY HIDDEN OBJECT NAMING TIME (sec)** | -0.003 | 0.001 | *0.002* | 725 | -0.01 | 0.001 | 0.114 | 725 | -0.002 | 0.001 | *0.032* | 725 | -0.001 | 0.001 | 0.402 | 725 |
| **PARTIALLY HIDDEN OBJECT NAMING ERRORS (n.)** | -0.06 | 0.008 | *0.000* | 762 | -0.02 | 0.009 | *0.012* | 762 | -0.04 | 0.008 | *0.000* | 762 | -0.02 | 0.009 | *0.047* | 762 |
| **POINTED OBJECTS NAMING** | -0.19 | 0.026 | *0.000* | 743 | -0.18 | 0.029 | *0.000* | 743 | -0.17 | 0.027 | *0.000* | 743 | -0.13 | 0.031 | *0.000* | 743 |

**Table S4.** Results of the regression analysis between object control skills sub-items and pre-literacy skills

|  | **Two-hand striking** | | |  | **Stationary bouncing** | | |  | **Catching** | |  |  | **Kicking** |  |  |  | **Overhand throwing** | | |  | |
| --- | --- | --- | --- | --- | --- | --- | --- | --- | --- | --- | --- | --- | --- | --- | --- | --- | --- | --- | --- | --- | --- |
|  | coef | SE | p | N | coef | SE | p | N | coef | SE | p | N | coef | SE | p | N | coef | SE | p | | N |
| **PRINTED LETTERS** | -0.05 | 0.017 | *0.002* | 772 | -0.06 | 0.015 | *0.000* | 772 | -0.05 | 0.016 | *0.002* | 772 | -0.03 | 0.018 | 0.114 | 772 | -0.05 | 0.018 | *0.014* | | 772 |
| **OBJECT NAMING TIME (sec)** | -0.02 | 0.002 | 0.375 | 733 | 0.001 | 0.002 | 0.573 | 733 | -0.003 | 0.002 | 0.079 | 733 | 0.001 | 0.002 | 0.503 | 733 | -0.00 | 0.002 | 0.964 | | 733 |
| **OBJECT NAMING ERRORS (n.)** | -0.04 | 0.026 | 0.139 | 730 | -0.03 | 0.022 | 0.217 | 730 | -0.04 | 0.025 | 0.076 | 730 | -0.08 | 0.026 | *0.003* | 730 | -0.08 | 0.027 | *0.006* | | 730 |
| **PARTIALLY HIDDEN OBJECT NAMING TIME (sec)** | 0.002 | 0.001 | 0.257 | 725 | -0.001 | 0.001 | 0.356 | 725 | -0.000 | 0.001 | 0.729 | 725 | -0.000 | 0.001 | 0.939 | 725 | 0.000 | 0.001 | 0.884 | | 725 |
| **PARTIALLY HIDDEN OBJECT NAMING ERRORS (n.)** | -0.02 | 0.009 | *0.024* | 762 | -0.04 | 0.008 | *0.000* | 762 | -0.029 | 0.009 | *0.002* | 762 | -0.04 | 0.010 | *0.000* | 762 | -0.02 | 0.011 | 0.090 | | 762 |
| **POINTED OBJECTS NAMING** | -0.18 | 0.032 | *0.000* | 743 | -0.18 | 0.027 | *0.000* | 743 | -0.20 | 0.029 | *0.000* | 743 | -0.15 | 0.033 | *0.000* | 743 | -0.13 | 0.034 | *0.000* | | 743 |
